# Association of *MRC-1* and *IL-28B* with the treatment outcome of hepatitis C: a case control study

**DOI:** 10.1186/1471-230X-14-113

**Published:** 2014-06-26

**Authors:** Cheng-Yuan Peng, Ter-Hsin Chen, Yun-Ping Lim, Fuu-Jen Tsai, Wei-Yong Lin, Wen-Ling Liao, Lei Wan

**Affiliations:** 1 Department of Internal Medicine, China Medical University Hospital, 40402 Taichung, Taiwan; 2 Graduate Institute of Veterinary Pathobiology, College of Veterinary Medicine, National Chung Hsing University, Taichung, Taiwan; 3 Department of Pharmacy, College of Pharmacy, China Medical University, 40402 Taichung, Taiwan; 4 School of Chinese Medicine, China Medical University, No. 91, Hsueh-Shih Road, 40402 Taichung, Taiwan; 5 Graduate Institute of Integrated Medicine, China Medical University, 40402 Taichung, Taiwan; 6 Department of Gynecology, China Medical University Hospital, 40447 Taichung, Taiwan; 7 Department of Biotechnology, Asia University, 41354 Taichung, Taiwan

**Keywords:** Hepatitis C virus, Standard of care treatment, Sustained virological response, Mannose receptor C, Interleukin-28B

## Abstract

**Background:**

The aim of this study was to evaluate whether polymorphisms of the mannose receptor C type 1 (*MRC-1*) and interleukin 28B (*IL-28B*) genes are associated with the treatment outcome of patients infected with hepatitis C virus genotypes 1 and 2 (HCV-1 and HCV-2, respectively) who are treated with peginterferon plus ribavirin (PEG-IFNα-RBV).

**Methods:**

We analyzed the association of the patients’ sustained viral responses (SVRs) to PEG-IFNα-RBV therapy with 2 single nucleotide polymorphisms (SNPs) in *MRC-1* and 3 SNPs in *IL-28B*. We selected patients infected with either HCV-1 (n = 265) or HCV-2 (n = 195) with or without SVR.

**Results:**

Among the *MRC-1* SNPs, rs691005 was found to be associated with SVR in HCV-1-infected patients (*P* < 0.0001). The *IL-28B* rs8099917 SNP was found to be associated with SVR in HCV-1- and HCV-2-infected patients (HCV-1, *P* < 0.0001; HCV-2, *P* = 0.002), while *IL-28B* rs955155 and rs10853728 SNPs were found to be associated with SVR in HCV-1-infected patients (*P* = 0.003) and HCV-2-infected patients (*P* = 0.02), respectively. We also identified an interaction between *MRC-1* rs691005 and *IL-28B* rs8099917 (*P* = 0.001). The C-T haplotype was shown to have a positive effect on SVR in HCV-1-infected patients (OR = 1.77, 95% CI = 1.2, 2.62), whereas the T-G haplotype was shown to have a negative effect on SVR in HCV-1-infected patients (OR = 0.28, 95% CI = 0.14, 0.58).

**Conclusions:**

These results suggest that SNPs of *IL-28B* and *MRC-1* can be used as genetic markers for predicting the outcome of PEG-IFNα-RBV treatment of HCV infections.

## Background

Pegylated interferon alpha (PEG-IFNα) in combination with ribavirin is the standard of care (SOC) recommended for treating chronic infections caused by the hepatitis C virus (HCV) [1]. Both the HCV and patient genotypes influence the effects of SOC treatment, thus leading to variations in treatment outcome [2,3]. SOC treatment of HCV genotype 2 (HCV-2) or 3 (HCV-3) infections has been shown to generate superior virological responses than SOC treatment of HCV-1 [4]. Among HCV-2/3 patients, the rates of rapid virological response (RVR) and sustained virological response (SVR) have been reported to be approximately 62–87% and 80%, respectively, whereas the SVR rate is only 50% in HCV-1 infected patients [4,5]. These findings indicate that HCV genotype-specific virological responses occur following SOC treatment [6]. In addition to virological responses, the diversity of host genetic factors plays an important role in treatment outcome. Genome-wide association studies have shown that single nucleotide polymorphisms (SNPs) in or near the interleukin-28B (*IL-28B*) gene are significantly associated with the treatment outcome for HCV-1-infected patients [3,7]. In addition, several studies have shown a significant association between *IL-28B* and SVR in HCV-2-infected patients [8]. However, the predictive value of these SNPs should be considered. For instance, the minor allele frequencies (MAFs) of the rs8099917 SNP are approximately 15.2% in Caucasian populations but only 6.5% in Chinese populations [3,8-10]. Thus, as most individuals carry the T/T genotype, which is associated with SVR, the potential predictive value of the rs8099917 T/T genotype might be misinterpreted. Therefore, it is important to find a more suitable marker for predicting the treatment outcome.

Recent insights into the complex mechanisms of HCV treatment outcome suggest that genetic variability in the genes encoding pattern recognition receptors (PRRs) such as Toll-like receptors (TLRs) plays a role in virological responses [11,12]. The mannose receptor (MR) is a PRR that binds to glycan structures containing mannose, fucose, and N-acetylglucosamine, which are found in the cell walls of several pathogenic microorganisms such as bacteria, parasites, yeasts, and viruses [13-16]. MR is a C-type lectin receptor that is predominantly expressed in macrophages and dendritic cells. MR acts as hepatitis B virus (HBV) surface antigen receptor, and it likely contributes to the impairment of dendritic cells involved in the inactivation of anti-viral responses by HBV [17]. Signaling through MR promotes Th1- or Th2-biased immune responses [18] and may be an important factor for determining the treatment outcomes of HCV-infected patients. MR also plays an important role in innate immunity. The MR C type 1 gene (*MRC-1*) is located on chromosome 10p12 and consists of 30 exons. Several reports have shown that *MRC-1* is associated with susceptibility to a subset of diseases, including asthma [19], sarcoidosis [20], and leprosy [21,22].

In the present study, we investigated the association of *MRC-1* and *IL-28B* SNPs with RVR and SVR in Taiwan Chinese patients undergoing PEG-IFNα-RBV treatment. Our results suggest that *MRC-1* is superior to *IL-28B* as a candidate gene for predicting the therapeutic outcomes of Taiwan Chinese patients infected with HCV-1 and HCV-2.

## Methods

### Patients

A total of 265 HCV-1 infected patients and 195 HCV-2 infected patients from China Medical University Hospital, Taichung, Taiwan, were enrolled. HCV infection diagnosis was based on elevation of serum transaminase levels for at least 6 months, serum anti-HCV-positivity, and detection of serum HCV RNA. Patients who infected with hepatitis B virus or human immunodeficiency virus were excluded. Patients received PEG-IFNα (weekly injections, 1.5 μg/kg body weight) and oral RBV (600 mg for < 60 kg, 800 mg for 60–80 kg, or 1,000 mg for > 80 kg per day) 48 weeks (HCV-1) or 24 weeks (HCV-2). The inform consent were received from all enrolled subjects. This study was approved by the Ethics Committee of China Medical University Hospital, Taichung, Taiwan, and was conducted according to the Declaration of Helsinki.

### HCV genotyping and RNA measurements

HCV genotyping was performed by reverse hybridization assay in accordance to the classification of Simmonds *et al*. (INNO LiPA HCV-II; Innogenetics, Gent, Belgium). Virological response was determined using a qualitative HCV RNA assays from Roche Diagnostics with a sensitivity of 30–50 IU/mL (HCV Amplicor™ 2.0, Roche Diagnostics, Branchburg, NJ). The HCV RNA levels are reported as IU/mL. Patients were defined as (1) rapid virological responders (RVRs, HCV RNA negative at week 4 of treatment), denoted as RVR (+), or (2) non-rapid virological responders (non-RVRs, HCV RNA positive at week 4 of treatment), denoted as RVR (−) or (3) sustained virological responder (SVR; HCV RNA undetectable at week 24 after the end of treatment), denoted as SVR (+); and (4) non-sustained virological responder (non-SVR; HCV RNA detected at week 24 after the end of treatment), denoted as SVR (−) according to the quantitative HCV RNA results. Therefore, all subjects were classified as RVR (+/−) or SVR (+/−).

### Genomic DNA extraction and genotyping

Genomic DNA was extracted from the peripheral blood from all participants by using a genomic DNA isolation kit (Genomic DNA kit; QIAGEN, Valencia, CA) according to the manufacturer’s instructions. All SNPs in *IL-28B* (rs955155, rs8099917, and rs10853728) and *MRC-1* (rs1926736 and rs691005) were genotyped using an allele-specific extension method and ligation assay according to the manufacturer’s instructions (Illumina, San Diego, CA).

### Statistical analysis

The association between each SNP and RVR and SVR was assessed by the χ^2^ test or Fisher exact test. Genotype and allele frequencies in RVR (+) and RVR (−) or in SVR (+) and SVR (−) subjects were compared, and odds ratios (ORs) with 95% confidence intervals (CIs) were determined by unconditional logistic regression. Age, body mass index (BMI), and viral load were estimated by the Mann–Whitney *U* test. The differences between genotypes and viral loads were estimated by the Kruskal–Wallis test. Haplotypes were derived from unphased genotype data using the Bayesian statistical method in the software program Phase 2.1 [23,24]. The multifactor dimensionality reduction (MDR) method (Dartmouth Medical School, Hanover, NH) was used to detect the locus-locus interaction models. The interaction dendrogram was built according to hierarchical clustering algorithm. All statistical analyses were conducted using SPSS statistical software (Version 20.0 for Windows, Chicago, IL). A *P* value less than 0.05 were considered statistically significant.

## Results

### Patients

A total of 265 HCV-1-infected and 195 HCV-2-infected patients were enrolled in this study. Of these, 61.5% of HCV-1-infected patients and 91.3% of HCV-2-infected patients exhibited SVR (Table 1). These results are consistent with previous reports demonstrating that compared to HCV-1-infected patients, HCV-2-infected patients showed superior virological responses. No gender-specific differences in SVR (+/−) were observed in HCV-1- or HCV-2-infected patients. Significant differences in SVR (+/−) with respect to age at entry and BMI were observed among HCV-1-infected patients (*P* = 0.002 and *P* = 0.03, respectively). The viral load at the beginning of treatment was significantly different between HCV-1-infected patients with and without SVR (*P* = 0.001; Table 1).

**Table 1:** Characteristics of the HCV genotype 1 and 2 infected patients with PEG-IFNα-RBV therapy

	**HCV genotype 1 (HCV-1)**				**HCV genotype 2 (HCV-2)**			
	**All**	**SVR (+)**	**SVR (−)**	***P *** **value**	**All**	**SVR (+)**	**SVR (−)**	***P *** **value**
**Number of patients**	265	163	102	-	195	178	17	-
**Sex (male/female)**	129/136	84/79	45/57	0.2	88/107	81/97	7/10	0.7
**Mean age ± SD**	52.17 ± 10.27	50.69 ± 10.60	54.52 ± 9.29	0.002^a^	51.62 ± 10.89	51.15 ± 11.16	56.47 ± 5.86	0.05^a^
**BMI (mean ± SD)**	24.6 ± 3.1	24.2 ± 2.9	25.2 ± 3.3	0.03^a^	24.5 ± 3.5	24.5 ± 3.6	24.5 ± 2.7	0.9^a^
**Viral load (×10**^**6**^**)**	12.1 ± 16.4	11.0 ± 16.5	13.9 ± 16.2	0.001^a^	11.0 ± 19.0	10.3 ± 17.8	18.4 ± 27.9	0.3^a^

SVR, sustained virological response; BMI, body mass index.

^a^Mann–Whitney U test.

All of the genotyped *IL-28B* SNPs (rs955155, rs8099917, and rs10853728) and *MRC-1* SNPs (rs1926736 and rs691005) were in Hardy–Weinberg equilibrium (HWE; *P* > 0.05), indicating that no population stratification bias or genotyping error existed. Information on SNPs, including chromosome position, HWE, and MAF, is listed in Additional file 1: Table S1.

### Association of IL-28B and MRC-1 SNPs with SVR to HCV-1 treatment

Three SNPs in *IL-28B* (rs955155, rs8099917, and rs10853728) and 2 SNPs in *MRC-1* (rs1926736 and rs691005) were examined. The genotype distributions of *IL-28B* rs955155 and rs8099917 were found to be associated with SVR in HCV-1-infected patients (*P* = 0.003 and *P* < 0.0001, respectively). The probability of the rs955155 T/T + C/T genotype achieving SVR was 0.28-fold lower than that of the C/C genotype (*P* = 0.003). The probability of the rs8099917 G/G + T/G genotype achieving SVR was 0.21-fold lower than that of the T/T genotype (*P* < 0.0001; Table 2). The allele frequencies of *IL-28B* rs955155 and rs8099917 were found to be associated with SVR in HCV-1-infected patients (*P* = 0.007 and *P* < 0.0001, respectively; Additional file 1: Table S2). The *MRC-1* rs691005 genotype was significantly associated with SVR (*P* < 0.0001). Patients with the rs691005 C/C + C/T genotype had a 2.77-fold higher probability of achieving SVR than those with the T/T genotype (Table 2). In addition, the *MRC-1* rs691005 allele was associated with SVR in HCV-1-infected patients (*P* = 0.01) (Additional file 1: Table S2).

**Table 2:** **Genotype frequencies of *MRC1 *** **and *IL28B *** **single nucleotide polymorphisms in HCV-1 infected PEG-IFNα-RBV therapy patients with and without SVR in Taiwan Chinese population**

***MRC1***					***IL28B***				
**Genotype**	**SVR (+)**	**SVR (−)**	***P***	**OR (95% CI)**	**Genotype**	**SVR(+)**	**SVR (−)**	***P***	**OR (95% CI)**
	**N (%)**	**N (%)**				**N (%)**	**N (%)**		
**(*MRC1*) rs1926736**					**(*IL28B*) rs955155**				
C/C	57 (35.0)	31 (30.4)		0.97 (0.49, 1.92)	C/C	155 (95.1)	86 (84.3)		1
C/T	66 (40.5)	50 (49.0)		0.69 (0.36, 1.32)	C/T	7 (4.3)	16 (15.7)		0.24 (0.1-0.61)
T/T	40 (24.5)	21 (20.6)	0.39	1	T/T	1 (0.6)	0 (0.0)	0.005*	-
C/C + C/T	123 (75.5)	81 (79.4)	0.46	0.80 (0.44, 1.45)	T/T + C/T	8 (4.9)	16 (15.7)	0.003*	0.28 (0.11-0.67)
**(*MRC1*) rs691005**					**(*IL28B*) rs8099917**				
C/C	13 (8.0)	12 (11.8)		1.16 (0.49, 2.75)	T/T	151 (92.6)	74 (72.5)		1
C/T	93 (57.1)	29 (28.4)		3.43 (1.98, 5.96)	T/G	11 (6.7)	28 (27.5)		0.19 (0.09-0.41)
T/T	57 (34.9)	61 (59.8)	<0.0001*	1	G/G	1 (0.7)	0 (0.0)	<0.0001*	-
C/C + C/T	106 (65.1)	41 (40.2)	<0.0001*	2.77 (1.66, 4.61)	G/G + T/G	12 (7.4)	28 (27.5)	<0.0001*	0.21 (0.1-0.44)
					**(*IL28B*) rs10853728**				
					C/C	115 (71.0)	66 (65.3)		1
					C/G	47 (29.0)	35 (34.7)	0.34	0.77 (0.45-1.31)
					G/G	0 (0.0)	0 (0.0)	-	-
					G/G + C/G	47 (29.0)	35 (34.7)	0.34	0.77 (0.45-1.31)

*Abbreviations*: SNP, single nucleotide polymorphism; SVR, sustained virological response; OR, odds ratio; CI, confidence interval.

Genotype frequencies were determined by χ^2^ test using 3 × 2 or 2 × 2 tables as appropriate. Odds ratios and 95% CI per genotype were estimated by applying unconditional logistic regression. The *P* values less than 0.05 were considered significant, and are denoted with an asterisk.

### Association of IL-28B and MRC-1 SNPs with SVR to HCV-2 treatment

Among HCV-2-infected patients, none of the *MRC-1* SNP genotypes was found to correlate with SVR (Table 3). However, the genotype distribution of *MRC-1* rs1926736 was significantly different among HCV-2-infected patients with respect to RVR (data not shown). We also found that the *MRC-1* rs691005 allele was associated with SVR in HCV-2-infected patients (P = 0.02; Additional file 1: Table S2). The *IL-28B* rs8099917 and rs10853728 SNPs were found to be associated with SVR in HCV-2-infected patients (*P* = 0.002 and *P* = 0.02, respectively). The probability of the rs8099917 G/G + G/T genotype achieving SVR was 0.21-fold lower than that of the T/T genotype (*P* = 0.002). Similarly, the rs10853728 G/G + C/G genotype showed a 0.29-fold lower probability of achieving SVR than the C/C genotype (*P* = 0.02; Table 3). The allele frequencies of rs8099917 and rs10853728 were significantly associated with SVR (*P* = 0.003 and *P* = 0.03, respectively; Additional file 1: Table S2).

**Table 3:** **Genotype frequencies of *MRC1 *** **and *IL28B *** **single nucleotide polymorphisms in HCV-2 infected PEG-IFNα-RBV therapy patients with and without SVR in Taiwan Chinese population**

***MRC1***					***IL28B***				
**Genotype**	**SVR (+)**	**SVR (−)**	***P***	**OR (95% CI)**	**Genotype**	**SVR(+)**	**SVR (−)**	***P***	**OR (95% CI)**
	**N (%)**	**N (%)**				**N (%)**	**N (%)**		
**(*MRC1*) rs1926736**					**(*IL28B*) rs955155**				
C/C	50 (28.1)	3 (17.6)		3.23 (0.75, 13.84)	C/C	154 (86.5)	12 (70.6)		1
C/T	97 (54.5)	8 (47.1)		2.35 (0.76, 7.29)	C/T	24 (13.5)	5 (29.4)	0.08	0.37 (0.12-1.16)
T/T	31 (17.4)	6 (35.3)	0.2	1	T/T	0 (0.0)	0 (0.0)	-	-
C/C + C/T	147 (82.6)	11 (64.7)	0.07	2.59 (0.89, 7.52)	T/T + C/T	24 (13.5)	5 (29.4)	0.08	0.37 (0.12-1.16)
**( *MRC1 *) rs691005**					**( *IL28B *) rs8099917**				
C/C	16 (9.0)	4 (23.5)		0.19 (0.04, 0.82)	T/T	155 (87.1)	10 (58.8)		1
C/T	76 (42.7)	9 (53.0)		0.39 (0.12, 1.33)	T/G	23 (12.9)	7 (41.2)	0.002*	0.21 (0.07-0.61)
T/T	86 (48.3)	4 (23.5)	0.06	1	G/G	0 (0.0)	0 (0.0)		-
C/C + C/T	92 (51.7)	13 (76.5)	0.05	0.33 (0.10, 1.05)	G/G + T/G	23 (12.9)	7 (41.2)	0.002*	0.21 (0.07-0.61)
					**( *IL28B *) rs10853728**				
					C/C	116 (65.2)	6 (35.3)		1
					C/G	62 (34.8)	11 (64.7)	0.02*	0.29 (0.1-0.83)
					G/G	0 (0.0)	0 (0.0)	-	-
					G/G + C/G	62 (34.8)	11 (64.7)	0.02*	0.29 (0.1-0.83)

*Abbreviations*: SNP, single nucleotide polymorphism; SVR, sustained virological response; OR, odds ratio; CI, confidence interval.

Genotype frequencies were determined by χ^2^ test using 3 × 2 or 2 × 2 tables as appropriate. Odds ratios and 95% CI per genotype were estimated by applying unconditional logistic regression. The *P* values less than 0.05 were considered significant, and are denoted with an asterisk.

### Multifactor dimensionality reduction (MDR) analysis

**Figure 1:**
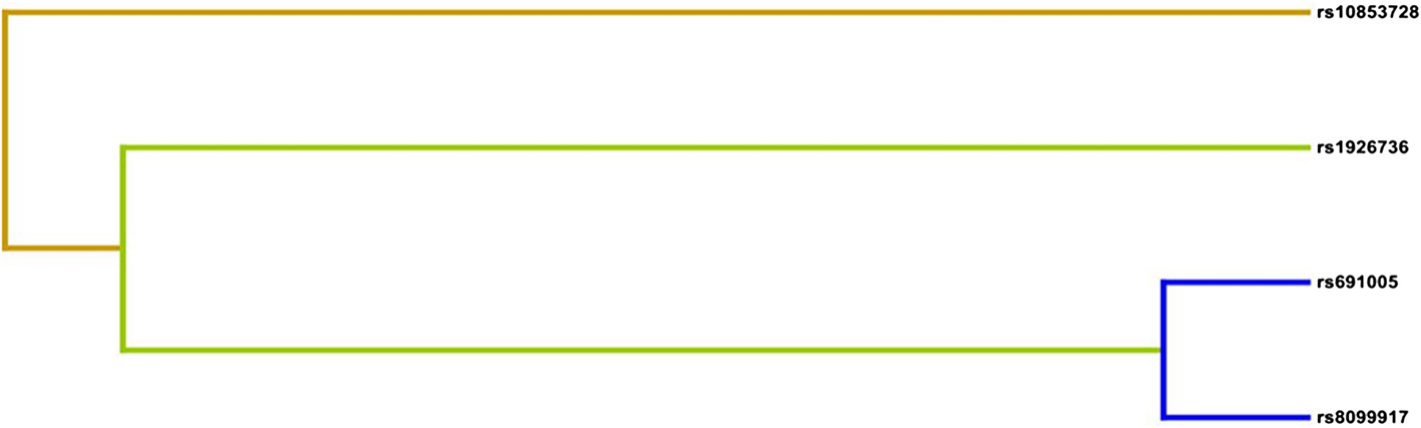
**Interaction dendrogram.** A strong interaction between *MRC-1* and *IL-28B* in the treatment outcome of HCV genotype 1 patients treated with PEG-IFNα-RBV is illustrated. The location of the longitudinal connecting bars indicates the strength of the dependence (left is weaker and right is stronger). Hierarchical cluster analysis with averaged linkages placed rs691005 and rs8099917 on the same branch.

We used MDR analysis to identify the best interaction models among the 5 *MRC-1* and *IL-28B* SNPs analyzed in this study (Table 4). In HCV-1-infected patients with SVR, we found a significant association with the 1-locus model (*MRC-1* rs691005; *P* = 0.01), 2-locus model (*MRC-1* rs691005 and *IL-28B* rs8099917; *P* = 0.001), 3-locus model (*IL-28B* rs1926736, *MRC-1* rs691005, and *IL-28B* rs8099917; P = 0.01), and 4-locus model (*IL-28B* rs1926736, *MRC-1* rs691005, *IL-28B* rs8099917, and *IL-28B* rs10853728; *P* = 0.01). No significant interactions were observed between *MRC-1* and *IL-28B* SNPs in HCV-2-infected patients with SVR. Based on the interaction dendrogram (Figure 1), a strong synergistic effect was observed between *MRC-1* rs691005 and *IL-28B* rs8099917.

**Table 4:** Summarizing multifactor dimensionality reduction (MDR) models for gene-gene interaction in HCV treatment outcome

**Number of factors**	**Best candidate models**	**Testing accuracy (%)**	***P *** value*	**Cross-validation consistency**
	**HCV-1**			
1	rs691005	64.3	0.01	10/10
2	rs691005 rs8099917	62.7	0.001	6/10
3	rs1926736 rs691005 rs8099917	61.3	0.01	6/10
4	rs1926736 rs691005 rs8099917 rs10853728	61.2	0.01	7/10
	**HCV-2**			
1	rs10857328	58.8	0.2	6/10
2	rs691005 rs8099917	70.01	0.06	10/10
3	rs1926736 rs691005 rs8099917	58.9	0.6	9/10
4	rs1926736 rs691005 rs8099917 rs10853728	49.2	0.96	10/10

*The P values less than 0.05 were considered significant.

### Frequencies of MRC-1 and IL-28B haplotypes

The *MRC-1* rs691005 and *IL-28B* rs8099917 haplotypes were analyzed to investigate a potential gene–gene interaction. A global test revealed a significant association with SVR in HCV-1-infected patients (*P* < 0.0001). The T-G haplotype was significantly inversely associated with SVR (OR = 0.28, 95% CI = 0.14–0.58; Table 5), whereas the C-T haplotype was significantly associated with SVR (OR = 1.77, 95% CI = 1.2–2.62; Table 5). To determine whether independent factors affected the outcome of PEG-IFNα-RBV therapy, multivariate logistic regression analysis was performed with respect to C − T/non-C − T diplotypes, gender, age, BMI, and HCV RNA levels (low/high). The HCV RNA level was considered low when the virus titer was found to be less than 100 KIU/ml by Amplicor-monitor assay. This analysis revealed that the C − T diplotype was independently associated with the outcome of PEG-IFNα-RBV therapy (*P <* 0.0001 OR = 2.96, 95% CI = 1.73, 5.06; Table 6). Furthermore, the data indicate that age and BMI also act together to influence the outcome of PEG-IFNα-RBV treatment (*P* = 0.01, OR = 0.96, 95% CI = 0.94, 0.99 and *P* = 0.048, OR = 0.914, 95% CI = 0.84, 1, respectively).

**Table 5:** Haplotype frequency of rs691005 and rs8099917 among HCV-1 infected PEG-IFNα-RBV therapy patients with and without SVR in Taiwan Chinese population

rs691005*	rs8099917*	**Positive (%)**	**Negative (%)**	***P *** value**	Odds Ratio (95% CI***)
C	G	1 (0.31)	4 (1.98)		0.15 (0.02-1.37)
C	T	118 (36.2)	49 (24.26)		1.77 (1.2-2.62)
T	G	12 (3.68)	24 (11.88)		0.28 (0.14-0.58)
T	T	195 (59.82)	125 (61.88)	< 0.0001	0.92 (0.64-1.31)

*The haplotypes were identified by the Bayesian statistical method available in the program Phase 2.1.

**The Pearson χ^2^ (5 × 2 table) was performed to obtain the *P* value. A *P* value of < 0.05 was considered as statistically significant.

***CI = confidence interval.

**Table 6:** Predictive factors associated independently with the SVR to PEG-IFNα-RBV therapy in HCV-1 infected patients by multivariate logistic regression analysis

**Parameter**	***P *** **value**	**OR (95% CI)**
Diplotype^§^ (C − T/non-C − T)	< 0.0001*	2.96 (1.73, 5.06)
Gender (males/females)	0.4	0.77 (0.45, 1.34)
Age	0.01*	0.96 (0.94, 0.99)
BMI	0.05	0.91 (0.84, 1.00)
HCV-RNA level^δ^ (low/high)	0.07	7.41 (0.89, 62.0)

*Abbreviation*: CI, confidence interval; BMI: body mass index.

^§^The order of the SNPs comprising the *IL28B* and *MRC1* diplotype was rs691005 and rs8099917.

^δ^Low HCV-RNA level: <100 KIU/ml by Amplicor-monitor assay. Odds ratios (OR) of having a SVR to PEG-IFNα-RBV therapy were calculated. *P* values less than 0.05 were considered statistically significant, and are denoted with an asterisk.

## Discussion and conclusions

In this study, we examined the association of HCV treatment efficacy with *MRC-1* and *IL-28B*. In addition to well-known loci on the *IL-28B* gene, SNPs located in *MRC-1* (rs1926736 and rs691005) were analyzed, and were shown to have a significant association with the outcome of HCV treatment using PEG-IFNα and ribavirin. Our results are consistent with other reports showing that *IL-28B* rs8099917 is associated with RVR and SVR in HCV-1- and HCV-2-infected patients. Our sample groups are comparable with those of previous studies; however, in most of these studies, the MAF of rs8099917 is generally lesser than 10% among Asian populations and greater than 15% among Caucasian populations [9,10,20]. We included *IL-28B* SNPs in our analysis to verify our sample quality, and we found that they generated results that are consistent with those of previous studies with respect to their association with HCV treatment outcome. To identify another useful prediction marker for the treatment outcome of HCV, we performed genotyping analyses of the rs1926736 and rs691005 SNPs in the *MRC-1* gene. *MRC-1* rs691005 was significantly associated with SVR in HCV-1-infected patients, and *MRC-1* rs1926736 was significantly associated with RVR in HCV-2-infected patients (data not shown). The MAFs for rs1926736 and rs691005 were 45.3% and 33.3%, respectively. The *MRC-1* rs691005 C/C + C/T genotype had a 2.77-fold higher probability of acquiring SVR than the T/T genotype. These results indicate that *IL-28B* and *MRC-1* are good predictors of PEG-IFNα-RBV treatment outcome for patients infected with HCV.

Several studies have suggested that dendritic cells can be infected with HCV [25-28]. C-type lectins play an important role in the receptor-mediated endocytosis of dendritic cells for T-cell presentation/activation. Several C-type lectins specific to mannosylated antigens are expressed by dendritic cells, such as langerin (CD207), MRC-1 (CD206), DEC-205 (CD205), and DC-specific intercellular adhesion molecule 3-grabbing nonintegrin (DC-SIGN; CD209) [29]. DC-SIGN has been shown to be important in the infection of dendritic cells by Ebola [30] and dengue viruses [31], which, like HCV, are members of the Flaviviridae family. Moreover, recombinant HCV envelope glycoprotein 2 (E2) and HCV pseudotype particles (HCVpps) have been shown to bind to DC-SIGN on dendritic cells [13,32]. Thus, blocking C-type lectins with mannan might reduce the binding of HCV-like particles to dendritic cells. However, blocking DC-SIGN with monoclonal antibodies was not sufficient to inhibit the binding of HCV-like particles to dendritic cells, indicating that other mannose receptors may participate in this process [27].

A number of studies have shown that genetic variants near *IL-28B* are associated with the outcome of treating HCV infections with PEG-IFNα-RBV. In the present study, we found that carriers of rs8099917 G variants (T-G + G-G) had a significantly higher risk of not achieving SVR. These results corroborate reports from China [9] and Japan [33], and confirm that *IL28B* rs8099917 is associated with SVR in different ethnic groups. The advantageous T allele of rs8099917 is present at a significantly higher frequency (97.2% in SVR(+) patients in this study) in Asian populations than in populations of African and Caucasian ancestry; this may explain the ethnic differences in SVR rates for IFN-based therapy among Asians, Europeans, and Africans. The current difficulties in evaluating the success rate of the IFN-based treatment may be alleviated by the findings of this study. In the present study, we found that *MRC-1* rs691005 could be used as another marker to predict the treatment outcome of treatment of HCV-1 infections. In order to achieve the most cost-effective treatment and reduce the possibility of serious side effects due to long treatment courses, predicting the treatment outcome of IFN-based therapy must be emphasized.

Customized therapy for HCV infections based on the patient’s genotype and treatment responses is becoming possible. In Taiwan, the standard duration of PEG-IFNα-RBV therapy against HCV is 24 weeks. However, some patients may not exhibit SVR at the end of this treatment, but may achieve SVR by increasing the treatment time. Therefore, we suggest that HCV-1- and HCV-2-infected patients carrying *IL28B* and *MRC-1* low-response alleles/genotypes may benefit from longer antiviral treatments. Variations in the human genome explain some of the difference observed in therapeutic efficacy. The combination of clinical information, including HCV genotypes, HCV viral load, cellular and viral gene expression profiles, and host genetic variations, would be useful in determining the appropriate treatment dose and duration, which could potentially minimize the side effects of drugs, improve the quality of life of patients, and reduce costs. Here, we report another human genome variation that can facilitate the prediction of treatment outcome. In conclusion, the present study indicates that genotyping of *IL28B* and *MRC-1* SNPs may provide novel guidelines for determining optimal treatment regimens for HCV infections.

## Competing interests

The authors declare no competing interests.

## Authors’ contributions

YPL, THC, and CYP designed and carried out the majority of the study. FJT, WLL, and WYL participated in clinical data and information collection. LW conceived and supervised the project and reviewed the manuscript. All authors contributed to and approved the final manuscript by providing constructive suggestions.

## Pre-publication history

The pre-publication history for this paper can be accessed here:

http://www.biomedcentral.com/1471-230X/14/113/prepub

## Supplementary Material

Additional file 1: Table S1 Five single nucleotide polymorphisms in the *MRC1* and *IL28B* gene identified from 265 HCV-1 and 195 HCV-2 infected PEG-IFNα-RBV therapy patients with or without SVR in Taiwan Chinese population. **Table S2.** Allele frequencies of *MRC1* and *IL28B* single nucleotide polymorphisms in HCV-1 and HCV-2 infected PEG-IFNα-RBV therapy patients with and without SVR in Taiwan Chinese population. Click here for file
