# Supplementary material for: Association of MRC-1 and IL-28B with the treatment outcome of hepatitis C: a case control study
Source: BMC Gastroenterol. 2014 Jun 26;14:113. doi: 10.1186/1471-230X-14-113 (PMC4098956; doi:10.1186/1471-230X-14-113)
Supplement: Additional file 1: Table S1 — Five single nucleotide polymorphisms in the MRC1 and IL28B gene identified from 265 HCV-1 and 195 HCV-2 infected PEG-IFNα-RBV therapy patients with or without SVR in Taiwan Chinese population. Table S2. Allele frequencies of MRC1 and IL28B single nucleotide polymorphisms in HCV-1 and HCV-2 infected PEG-IFNα-RBV therapy patients with and without SVR in Taiwan Chinese population. [file 1471-230X-14-113-S1.docx]

**Supplementary Tables**

**Additional file 1:** **Table S1. Five single nucleotide polymorphisms in the *MRC1* and *IL28B* gene identified from 265 HCV-1 and 195 HCV-2 infected PEG-IFNα-RBV therapy patients with or without SVR in Taiwan Chinese population.**

| Gene | SNPs | Position  in gene | Chromosome position | Alleles  (1/2) | HCV-1 | | | HCV-2 | | |
| --- | --- | --- | --- | --- | --- | --- | --- | --- | --- | --- |
|  |  |  |  |  | HWE  (*P* value) | MAF | | HWE  (*P* value) | MAF | |
|  |  |  |  |  |  | SVR (+) | SVR (-) |  | SVR (+) | SVR (-) |
| *MRC1* | rs1926736 | Exon 7 | Chr10:17891705 | C/T | 0.0629 | 0.4479 | 0.4510 | 0.3122 | 0.4466 | 0.4118 |
| *MRC1* | rs691005 | Downstream | Chr10:17962845 | C/T | 0.4841 | 0.3650 | 0.2598 | 1 | 0.3034 | 0.5000 |
| *IL28B* | rs955155 | Downstream | Chr19:39729479 | C/T | 0.4503 | 0.0276 | 0.0784 | 0.6051 | 0.0674 | 0.1471 |
| *IL28B* | rs8099917 | Promoter | Chr19:39743165 | T/G | 1 | 0.0399 | 0.1373 | 0.6085 | 0.0646 | 0.2059 |
| *IL28B* | rs10853728 | Promoter | Chr19:39745146 | C/G | 0.2693 | 0.1451 | 0.1733 | 0.0791 | 0.1742 | 0.3235 |

**Additional file 1:** **Table S2. Allele frequencies of *MRC1* and *IL28B* single nucleotide polymorphisms in HCV-1 and HCV-2 infected PEG-IFNα-RBV therapy patients with and without SVR in Taiwan Chinese population.**

| **HCV-1** | | | | | **HCV-2** | | | | |
| --- | --- | --- | --- | --- | --- | --- | --- | --- | --- |
| **SNP ID** | **SVR (+)**  **N (%)** | **SVR (-)**  **N (%)** | ***P*** | **OR (95% CI)** | **SNP ID** | **SVR (+)**  **N (%)** | **SVR (-)**  **N (%)** | ***P*** | **OR (95% CI)** |
| **(*MRC1*) rs1926736** | | | | | **(*MRC1*) rs1926736** | | | | |
| C allele | 180 (55.2) | 112 (54.9) |  | 1.01 (0.71, 1.44) | C allele | 197 (55.3) | 14 (41.2) |  | 1.77 (0.87, 3.62) |
| T allele | 146 (44.8) | 92 (45.1) | 0.9438 | 1 | T allele | 159 (44.7) | 20 (58.8) | 0.1134 | 1 |
| **(*MRC1*) rs691005** | | | | | **(*MRC1*) rs691005** | | | | |
| C allele | 119 (36.5) | 53 (26.0) |  | **1.64 (1.11, 2.41)** | C allele | 108 (30.3) | 17 (50.0) |  | **0.44 (0.21, 0.89)** |
| T allele | 207 (63.5) | 151 (74.0) | **0.0118** | 1 | T allele | 248 (69.7) | 17 (50.0) | **0.0189** | 1 |
| **(*IL28B*) rs955155** | | | | | **(*IL28B*) rs955155** | | | | |
| C allele | 317 (97.2) | 188 (92.2) |  | **3.00 (1.30, 6.92)** | C allele | 332 (93.3) | 29 (85.3) |  | 2.39 (0.85, 6.72) |
| T allele | 9 (2.8) | 16 (7.8) | **0.0072** | 1 | T allele | 24 (6.7) | 5 (14.7) | 0.0908 | 1 |
| **(*IL28B*) rs8099917** | | | | | **(*IL28B*) rs8099917** | | | | |
| T allele | 313 (96.0) | 176 (86.3) |  | **3.83 (1.93, 7.59)** | T allele | 333 (93.5) | 27 (79.4) |  | **3.75 (1.48, 9.54)** |
| G allele | 13 (4.0) | 28 (13.7) | **4.45×10^-5^** | 1 | G allele | 23 (6.5) | 7 (20.6) | **0.0031** | 1 |
| **(*IL28B*) rs10853728** | | | | | **(*IL28B*) rs10853728** | | | | |
| C allele | 277 (85.5) | 167 (82.7) |  | 1.24 (0.77, 1.99) | C allele | 294 (82.6) | 23 (67.6) |  | **2.27 (1.05, 4.89)** |
| G allele | 47 (14.5) | 35 (17.3) | 0.3858 | 1 | G allele | 62 (17.4) | 11 (32.4) | **0.0329** | 1 |

^a^: contain one missing data in both SVR (+) and SVR (-) groups.

Abbreviations: SNP, single nucleotide polymorphism; SVR, sustained virological response; OR, odds ratio; CI, confidence interval.

Allele frequencies were determined by χ^2^ test using 2 × 2 tables. Odds ratios and 95% CI per allele were estimated by applying unconditional logistic regression. The *P* values less than 0.05 were considered significant.
